# Sepsis-Associated Acute Kidney Injury and 28-Day Mortality in Critically Ill Patients with Sepsis: A Retrospective Cohort Study

**DOI:** 10.3390/jcm15166171

**Published:** 2026-08-09

**Authors:** İsa Kılıç, Abdülmecit Yıldız

**Affiliations:** 1 Department of Anaesthesiology and Intensive Care, Ministry of Health, Bursa City Hospital, Nilüfer, Bursa 16310, Turkey; 2 Department of Nephrology, Bursa Uludag Universitesi Tıp Fakultesi, Nilüfer, Bursa 16059, Turkey; mecit@uludag.edu.tr

**Keywords:** sepsis-associated acute kidney injury, mortality, intensive care unit, sepsis

## Abstract

**Background/Objectives**: We aimed to analyse the development of acute kidney injury (AKI), its association with mortality, and mortality-associated factors in patients admitted to the intensive care unit (ICU) with sepsis. **Methods**: This retrospective cohort study was conducted on adult patients with sepsis admitted to the ICU of a tertiary-care hospital between January 2023 and December 2023. Demographic, clinical, and laboratory data within the first 24 h of admission to the ICU were recorded. These data were compared between patients with and without sepsis-associated acute kidney injury (SA-AKI). **Results**: Among 1163 admissions, 185 met the study criteria, and the incidence of SA-AKI was 45.4%. The overall 28-day mortality was 38.4%. Patients with SA-AKI had significantly higher mortality than those without SA-AKI (63.1% vs. 17.8%, *p* < 0.001). In the multivariable Cox regression analysis, SA-AKI was independently associated with a higher risk of 28-day mortality (HR = 2.87, 95% CI 1.51–5.45, *p* = 0.001). Among patients with SA-AKI, elevated lactate showed a borderline association with mortality (HR = 1.06 per mmol/L, 95% CI 1.00–1.13, *p* = 0.053); first-day vasopressor use was not independently associated with mortality after adjustment for illness severity. **Conclusions**: SA-AKI was independently associated with higher 28-day mortality. Within the SA-AKI subgroup, lactate showed only a borderline association with mortality, whereas first-day vasopressor use appeared to reflect illness severity.

## 1. Introduction

Acute kidney injury (AKI) occurs frequently in the critically ill population. Higher stages of AKI, in particular, carry an increased likelihood of death both in hospital and within the intensive care unit (ICU) [1].

Sepsis, a syndrome characterized by a dysregulated host response to infection and life-threatening organ dysfunction, is one of the main etiologic determinants of AKI in critically ill patients. It accounts for 45–70% of AKI cases in this population [1,2].

When set against sepsis without renal involvement or AKI of non-septic origin, sepsis-associated acute kidney injury (SA-AKI) carries a poorer outlook: mortality is higher, ICU stays are longer, and hospital admissions are more prolonged [1,3].

Globally, sepsis still contributes to almost one in five deaths (19.7%) [4]. Contemporary large epidemiological works have documented an SA-AKI frequency of 46.6% to 62.3% among septic ICU admissions and confirmed that its presence worsens both morbidity and short-term survival [5,6].

Following the 28th Acute Disease Quality Initiative (ADQI) consensus meeting in June 2022, SA-AKI has been formally described as a discrete clinical entity [7]. Yet, despite this updated framework, few published studies have applied the ADQI 28 criteria, and evidence on how the individual serum-creatinine and urine-output diagnostic components relate to short-term outcomes is still limited.

Our working hypothesis was that, when applied strictly, the ADQI-based SA-AKI definition would flag a group of septic ICU patients at greater risk of dying within 28 days than septic patients without SA-AKI.

Guided by that consensus document, we designed the present analysis to characterize the development of AKI, examine its association with mortality, and identify other mortality-associated clinical variables in septic ICU patients.

## 2. Materials and Methods

### 2.1. Study Design

We reviewed, retrospectively, adult admissions carrying a diagnosis of sepsis to the 51-bed ICU of a tertiary referral centre (Bursa City Hospital) over the twelve months of 2023 (1 January to 31 December). The local ethics committee cleared the protocol (reference 2024-5/4; approved 3 April 2024) and waived the need for individual written consent given the retrospective nature of the data.

### 2.2. Patients and Data Collection

All consecutive ICU admissions during the study window were screened, and adults aged 18 years or older with sepsis were retained. We removed from the analysis anyone on chronic dialysis for end-stage renal disease (ESRD), kidney transplant recipients, admissions lasting under 24 h, cases not fulfilling Sepsis-3, minors, patients transferred in from other ICUs, and postoperative or trauma admissions.

The following variables were pulled from our hospital’s electronic health record: age and sex; a comorbidity block covering diabetes mellitus (DM), hypertension (HT), chronic obstructive pulmonary disease (COPD) and the Charlson Comorbidity Index (CCI); the Simplified Acute Physiology Score II (SAPS II); the referring location (emergency room, hospital ward, or operating theatre); admission-day laboratory values (lactate, bicarbonate, and others); microbiological culture data and the isolated organism; the focus of infection (pulmonary, intra-abdominal, urinary, bloodstream, and so on); whether nephrotoxic medications vancomycin, aminoglycosides, non-steroidal anti-inflammatory drugs, angiotensin-converting-enzyme inhibitors, or angiotensin-receptor blockers were being administered around the time sepsis developed; ventilator use; requirement for inotropes; the timing of infection onset, AKI onset, and death (up to day 28); and total ICU length of stay (LOS). Vital signs (temperature, heart rate, mean arterial pressure) as well as hourly urine output were extracted from bedside nursing observation records held in the same electronic system (Bursa City Hospital Information Management System, Ceiba Health eClinics Platform, Sarıyer, İstanbul, Türkiye). Urine-output entries in the nursing record showed no gaps.

All-cause death within 28 days served as the primary endpoint. Secondary endpoints comprised in-hospital and ICU mortality, requirement for renal replacement therapy (RRT), ICU LOS, and total hospital LOS.

### 2.3. Definitions

In keeping with Sepsis-3, sepsis was flagged when organ dysfunction arose in the context of a confirmed or presumed infection [8]. Antibiotic initiation and the drawing of cultures were both taken as markers of clinically suspected infection, with the infection reference time set at whichever event came first. Cultures obtained for routine surveillance were disregarded. Sepsis onset was defined as the earliest calendar day showing a rise of at least two points in the Sequential Organ Failure Assessment (SOFA) score inside a fixed window running from 48 h before to 24 h after the infection reference time [8,9]. Any pre-existing SOFA score before that reference point was set at zero. Missing data for a given organ system contributed nothing to the SOFA total [10]. In sedated patients, the neurological component was likewise omitted [11]. Septic shock was recorded when a septic patient required a vasopressor and had a blood lactate above 2 mmol/L [12].

AKI diagnosis and staging (1–3) followed the 2012 Kidney Disease: Improving Global Outcomes (KDIGO) framework, using both creatinine and urine-output limbs [13]. Urine output was expressed per kilogram of the body weight documented at ICU admission. Six-, twelve-, and twenty-four-hour urine-output windows were reviewed by hand from the electronic charts to establish whether the KDIGO urine-output threshold had been crossed [14]. Applying the ADQI 28 consensus wording, the SA-AKI diagnosis was time-stamped at the earliest moment within the seven days after sepsis diagnosis at which either the creatinine or the urine-output KDIGO component was reached [7]. Patients whose AKI predated sepsis were not counted as SA-AKI cases.

Within the SA-AKI group we then split cases by the diagnostic criterion that had been met at any point during the episode: (1) urine output alone (UO-only); (2) creatinine alone (SCr-only); and (3) both criteria at any time (Both). This grouping was exploratory. Its aim was not to benchmark diagnostic approaches against one another but to identify clinically distinct subphenotypes with potentially different trajectories.

Where KDIGO thresholds were already met on the day of ICU admission, that day was labelled day 0 of AKI. Severity was recorded as the highest KDIGO stage attained on either the creatinine or the urine-output limb during the first seven days after ICU admission. Every included case had an indwelling urinary catheter; hourly measurements were complete and no data were lost. Chronic kidney disease (CKD) was accepted either by prior clinical documentation or by the presence of at least two estimated glomerular filtration rate (eGFR) readings below 60 mL/min/1.73 m^2^ separated by more than three months [15].

When a pre-admission creatinine value was on file, we took the lowest reading of the preceding twelve months as baseline. When no such value existed, the lowest creatinine observed during the ICU stay was substituted.

### 2.4. Statistical Analysis

The main analyses ran on IBM SPSS Statistics for macOS, version 30.0 (IBM Corp., Armonk, NY, USA). Selected additional analyses, including analyses based on scaled Schoenfeld residuals, time-varying Cox models, and bootstrap power estimates were conducted in Python 3.12.3 using lifelines version 0.30.3 and statsmodels version 0.14.6. Continuous variables were checked for normality with the Kolmogorov–Smirnov test together with skewness and kurtosis within ±2, and are reported either as mean ± SD or as median with interquartile range (IQR). Categorical fields are given as counts with percentages, presented as *n* (%).

Two-group comparisons of continuous data used, according to distribution, the Student’s *t*-test, the Welch’s *t*-test, or the Mann–Whitney U test. When contrasting the three AKI diagnostic patterns (SCr-only, UO-only, and SCr + UO), one-way ANOVA or the Kruskal–Wallis test was applied. Categorical variables were tested with the Pearson’s χ^2^ test or Fisher exact test, and Bonferroni corrections were applied to post hoc pairwise contrasts.

The impact of individual variables on 28-day mortality was quantified with Cox proportional hazards regression, run separately in the whole cohort and in the SA-AKI subset. Follow-up ran from the sepsis diagnosis until death, and cases still alive at day 28 were censored. To keep hazard ratios interpretable, age and SAPS II were entered per 10 units. Variables carried into the multivariable models were chosen a priori on clinical grounds. Multicollinearity was screened with variance inflation factors, and model discrimination was summarised by Harrell’s C statistic. The proportional hazards assumption was tested formally through scaled Schoenfeld residuals. Kaplan–Meier curves were compared using the log-rank test, and effect sizes are presented as hazard ratios (HRs) with 95% confidence intervals (CIs).

Three complementary sensitivity checks probed the robustness of the SA-AKI mortality link: a baseline-restricted model in which SA-AKI was counted only when present on ICU day 0; a landmark analysis anchored at day 7; and a Cox model in which SA-AKI entered as a time-varying covariate. A fourth sensitivity exercise re-imputed baseline creatinine for the seven cases (3.8%) lacking a documented pre-admission value using the Modification of Diet in Renal Disease (MDRD) equation with eGFR set at 75 mL/min/1.73 m^2^.

## 3. Results

### 3.1. Patient Characteristics

**Figure 1:**
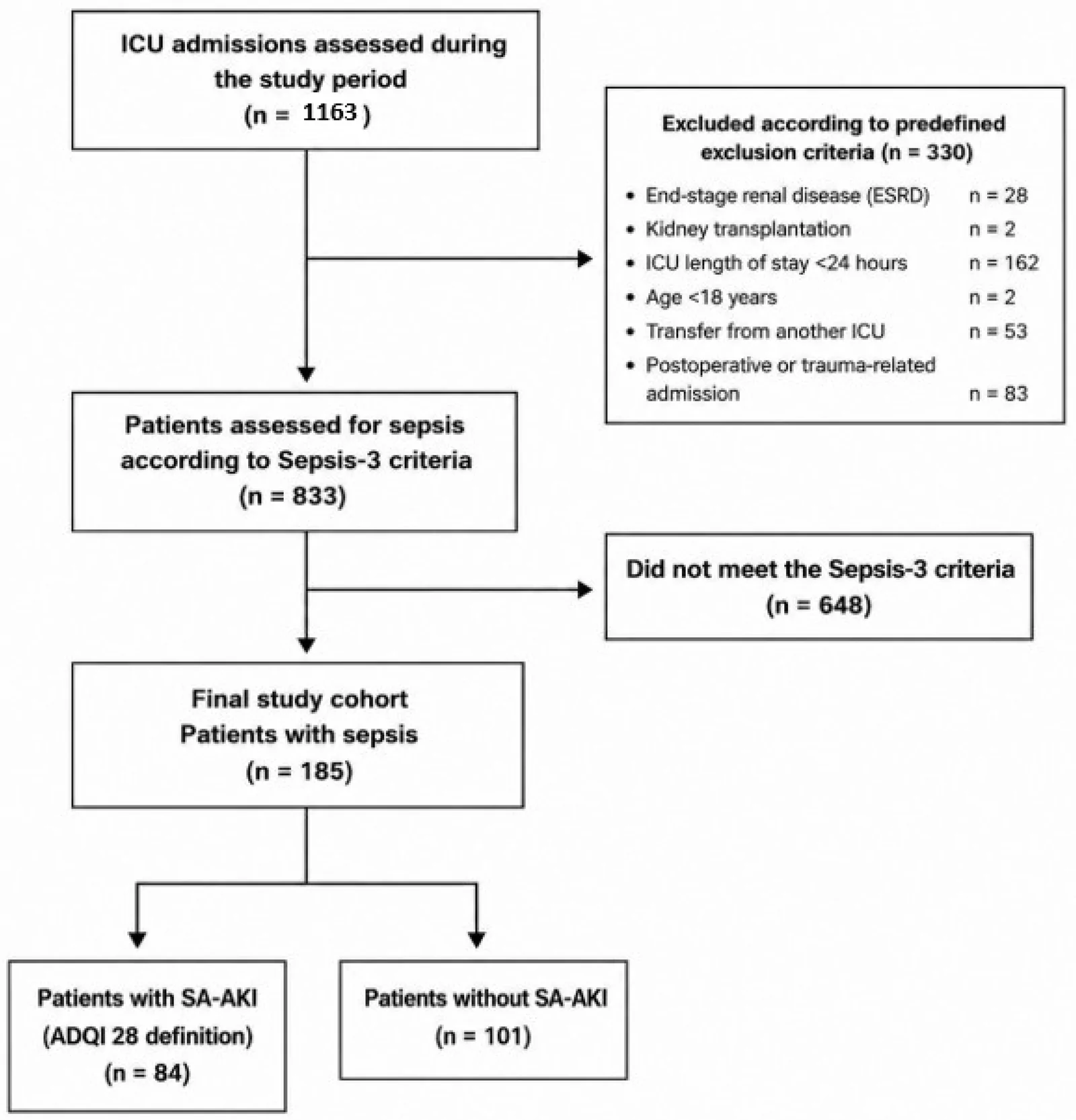
Flow diagram showing patient selection and formation of the study cohort. *ICU: intensive care unit, ESRD: end-stage renal disease, SA-AKI: sepsis-associated acute kidney injury, ADQI: Acute Disease Quality Initiative.*

Figure 1 summarises the flow of participants. Of the 1163 ICU admissions logged during the twelve months, 330 were removed on pre-defined exclusion grounds. The remaining 833 records were reviewed against Sepsis-3, and 648 did not qualify. The final analytic cohort therefore comprised 185 septic adults, split into 84 who met the ADQI 28 SA-AKI definition and 101 who did not (Figure 1).

Cases developing SA-AKI carried a heavier clinical load: septic shock, exposure to nephrotoxins, cardiovascular disease, malignancy, and CKD were all more common. CCI scores, admission lactate, baseline creatinine, and SAPS II were higher in this group, while platelet counts, arterial pH, bicarbonate, albumin, and mean arterial pressure were correspondingly lower (Table 1).

A documented pre-admission creatinine drawn from clinic records within twelve months of hospitalisation was retrievable for 178 patients (96.2%). For the seven (3.8%) without such a reading, the lowest ICU creatinine was substituted; a further sensitivity check re-derived baseline via the MDRD equation with eGFR fixed at 75 mL/min/1.73 m^2^ (Supplementary Table S1).

### 3.2. Incidence and Characteristics of SA-AKI

SA-AKI arose in 84 individuals (45.4% of the analytic cohort). Distribution across KDIGO categories was 20 cases (23.8%) at stage 1, 16 (19.0%) at stage 2, and 48 (57.1%) at stage 3. In terms of which limb of the definition triggered the label, 23 (27.4%) qualified through serum creatinine (SCr) only, 16 (19.0%) through urine output (UO) only, and 45 (53.6%) via both criteria (SCr + UO) (Table 2). Among the 84 patients with SA-AKI, 62 (73.8%) met the diagnostic criteria on ICU day 0; the remaining cases were first identified on day 1 (*n* = 19), day 2 (*n* = 1), or day 6 (*n* = 2). The median day of sepsis diagnosis was ICU day 0 (IQR, 0–0).

### 3.3. Clinical Outcomes According to SA-AKI and 28-Day Mortality

Compared to septic patients without kidney involvement, those with SA-AKI more often received vasopressors, mechanical ventilation, and RRT. Both ICU stay (median 11.0 [5.0–22.2] vs. 17.5 [8.0–36.2] days, *p* = 0.009) and total hospital stay (median 19.0 [10.0–33.2] vs. 25.5 [14.8–43.8] days, *p* = 0.011) were shorter in the SA-AKI group, a finding that should be interpreted alongside the high early mortality observed in the same group.

Overall, 71 of the 185 patients died within 28 days (38.4%) and 97 (52.4%) died before discharge. Both rates were markedly higher in SA-AKI cases (63.1% vs. 17.8% at 28 days, and 75.0% vs. 33.7% in hospital; *p* < 0.001 for each) (Table 2).

Outcome and microbiology data broken down by 28-day survival status appear in Supplementary Table S2. Among non-survivors, ICU and hospital stays were shorter, a pattern consistent with early death, whereas vasopressor, ventilator, and RRT requirements were more frequent. Klebsiella pneumoniae was also isolated more often among decedents.

### 3.4. Factors Associated with 28-Day Mortality

Non-survivors more frequently had SA-AKI, septic shock, nephrotoxin exposure, cancer diagnoses, CKD, day-1 RRT, and day-1 vasopressor requirements. Their admission lactate, SAPS II, and creatinine values ran higher, whereas albumin, platelet counts, and both daily and hourly urine outputs were lower (Table 3).

### 3.5. Univariable and Multivariable Cox Proportional Hazards Analyses of Factors Associated with 28-Day Mortality in the Overall Cohort

**Figure 2:**
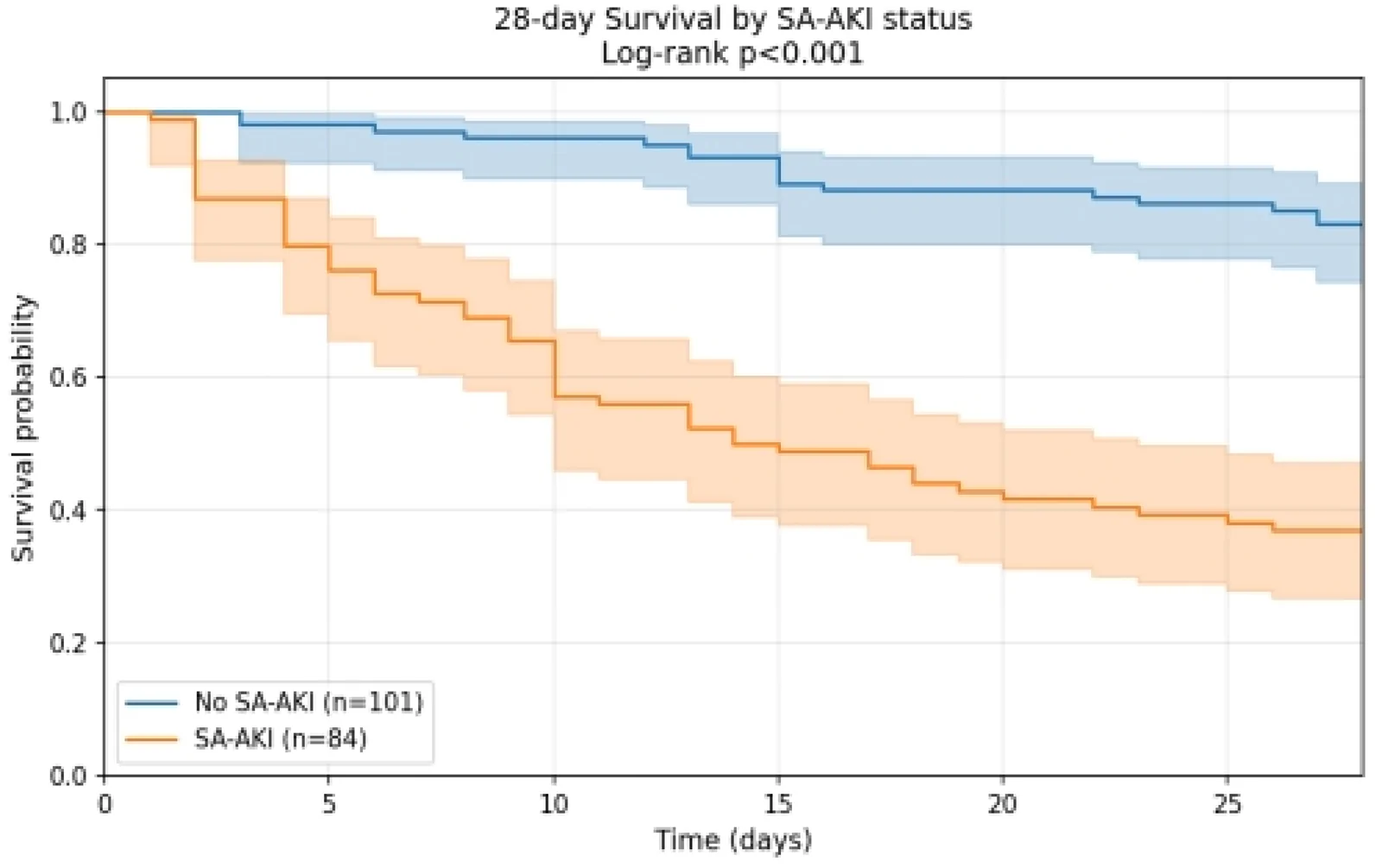
Kaplan–Meier 28-day survival by SA-AKI status. Twenty-eight-day survival probability among patients with and without sepsis-associated acute kidney injury (SA-AKI). Shaded areas represent 95% confidence intervals. Log-rank test *p* < 0.001.

Kaplan–Meier estimation, tested with the log-rank statistic, showed a clear separation of survival curves in favour of septic patients without kidney injury (*p* < 0.001) (Figure 2).

At the univariable level, the following variables all pointed toward higher 28-day mortality: SA-AKI, septic shock, exposure to nephrotoxic drugs, cancer, CKD, CCI, admission lactate, SAPS II, day-1 RRT, and day-1 vasopressor use; conversely, higher albumin tracked with better survival. The pre-specified multivariable model selected on clinical grounds carried SA-AKI, age, CCI, lactate, SAPS II, albumin, and day-1 vasopressor use. Centred variance inflation factors (VIFs) ranged from 1.20 to 2.53, well below the pre-specified threshold of 5, ruling out troublesome collinearity. Only SA-AKI was independently associated with 28-day mortality (HR = 2.87; 95% CI 1.51–5.45; *p* = 0.001); none of the remaining covariates reached significance, although SAPS II showed a borderline trend (HR = 1.18 per 10 points; 95% CI 0.98–1.40; *p* = 0.074). The model was fitted using data from 185 patients with 71 events, yielding a Harrell C statistic of 0.773 (Table 4).

Formal testing of the proportional hazards assumption via scaled Schoenfeld residuals is set out in Supplementary Table S3. It was satisfied for SA-AKI and the remaining covariates in the model; only albumin gave a borderline signal (*p* = 0.040).

### 3.6. Factors Associated with 28-Day Mortality in SA-AKI Patients: Univariable and Multivariable Cox Proportional Hazards Analyses

Restricting the analysis to SA-AKI cases and running univariable Cox regressions, higher 28-day mortality was tied to septic shock, malignancy, CKD, CCI, lactate, SAPS II, and day-1 vasopressor use. Nephrotoxin exposure sat at the border of significance (*p* = 0.051). Neither the AKI diagnostic pattern, the AKI stage, day-1 RRT, nor day-1 ventilation showed a signal. Once the pre-specified multivariable model (CCI, lactate, SAPS II, day-1 vasopressor, and the AKI diagnostic pattern) was fitted, no variable met the α = 0.05 threshold. Lactate did trend toward higher mortality but stopped short of significance (HR = 1.06 per 1 mmol/L; 95% CI 1.00–1.13; *p* = 0.053). Discrimination, measured by Harrell C, was 0.664 (Table 5).

In this subgroup model, scaled Schoenfeld testing flagged the proportional hazards assumption as violated for CCI (*p* = 0.035), SAPS II (*p* = 0.039), and the SCr + UO diagnostic pattern (*p* = 0.001) (Supplementary Table S3). Because these associations may vary over the 28-day follow-up, the subgroup model should be considered exploratory, and its adjusted hazard ratios should not be interpreted as constant associations over time.

### 3.7. Sensitivity Analyses

We checked the robustness of the SA-AKI–mortality link by comparing the primary Cox model against four complementary specifications (Supplementary Table S4 and Figure S1). In the primary model the adjusted HR for SA-AKI was 2.87 (95% CI 1.51–5.45; *p* = 0.001). Restricting exposure to SA-AKI present on ICU day 0 gave a similar estimate (*n* = 163; 57 events; HR = 2.86; 95% CI 1.42–5.74; *p* = 0.003). The day-7 landmark model produced HR = 2.82 (*n* = 159; 45 events; 95% CI 1.33–6.00; *p* = 0.007), and the time-varying Cox specification returned HR = 2.71 (95% CI 1.44–5.10; *p* = 0.002). Substituting MDRD-derived values for the missing baseline creatinines likewise preserved the association (*n* = 185; HR = 2.83; 95% CI 1.49–5.41; *p* = 0.002).

Across all five specifications, the SA-AKI hazard ratios clustered tightly between roughly 2.7 and 2.9 and every one reached statistical significance. Neither adjustment for immortal-time bias nor an alternative baseline-creatinine handling erased the link.

### 3.8. Subgroup Analysis: AKI Diagnostic Patterns (Exploratory)

Comparisons across the three diagnostic patterns are given in Supplementary Tables S5 and S6. The groups differed significantly in septic-shock frequency, lactate level, SAPS II, day-1 vasopressor requirement, and AKI stage. The SCr + UO subset (fulfilling both KDIGO limbs) topped every one of these variables, and stage 3 AKI was concentrated there. Neither 28-day nor in-hospital mortality, however, differed among the patterns. Taking UO-only as the referent in an unadjusted Cox model, neither SCr-only nor SCr + UO showed a significant association with 28-day mortality.

These pattern-level analyses are exploratory. Small cell sizes (16–45 cases per pattern) and wide confidence intervals prevent us from drawing firm conclusions about mortality differences between patterns. They should be treated as hypothesis-generating and re-tested in larger series.

## 4. Discussion

A pooled analysis spanning 189 studies has shown that SA-AKI frequency depends strongly on the definition applied: rates were as low as 26% when only RRT-requiring cases were counted, but climbed toward 52% under KDIGO criteria [16]. Our incidence (45.4%) aligns closely with the 46.6% reported by Takeuchi and colleagues, whereas larger multicentre efforts by White, Song, and others have reported lower figures most probably because of differences in patient mix and methodological choices [5,6,14]. On severity, stage 3 dominated our SA-AKI group, echoing an earlier observation by Song and colleagues. Also worth noting: in about three of every four septic patients, SA-AKI was already established on ICU day 0, reinforcing prior reports that renal injury tends to emerge very early and is usually identifiable at the point of ICU entry [5,14].

SA-AKI is widely recognized as an important clinical marker in patients with sepsis and is associated with increased mortality [6]. A meta-analysis of 47 observational reports totalling 55,911 septic patients placed 28-day mortality at 36.67%. In our SA-AKI subset, mortality ran roughly 10 percentage points above that reference figure. Our data confirm the association between SA-AKI and increased mortality in the septic population and suggest that SA-AKI reflects a heavier disease burden. That said, the pooled figure rested on only four studies, and the wider literature on this precise question remains thin; substantial heterogeneity among short-term mortality estimates therefore likely exists, and comparisons must be drawn cautiously [17]. Newer mechanistic work argues that systemic inflammation, endothelial injury, and inter-organ signalling may amplify immune activation and drive renal damage. Even though this framework has largely been developed in the CKD context, it supports the broader view that kidney injury in critical illness is not simply a hemodynamic phenomenon but a multifactorial one [18].

Hemodynamic instability strongly shapes prognosis in SA-AKI. Older evidence positioned norepinephrine as improving organ perfusion and possibly cutting mortality in septic shock [19,20], yet more recent series have suggested that vasoactive drug use in septic shock may in fact accompany higher mortality. In Takeuchi et al., SA-AKI patients with concomitant septic shock had a greater comorbidity burden, higher illness severity scores, and poorer in-hospital survival [6]; in Zeng et al., norepinephrine use was independently associated with 28-day mortality among elderly patients with SA-AKI [21]. In our own data, day-1 vasopressor use was strongly linked to 28-day mortality on univariable analysis, but this signal weakened sharply on multivariable adjustment and disappeared statistically, a similar pattern held in the SA-AKI subset. These observations should not be read as direct evidence that norepinephrine kills patients. Rather, the need for a vasopressor probably functions as a clinical proxy for the underlying severity of shock and hemodynamic derangement. Experimental data support this interpretation: in awake sheep, Calzavacca and colleagues showed that norepinephrine can lower medullary oxygenation without meaningfully changing renal blood flow, illustrating that renal effects of vasoactive drugs are not captured by systemic pressure or renal blood flow alone [22]. Lankadeva and colleagues extended this finding by showing similar medullary hypoxia during norepinephrine resuscitation in an ovine septic shock model [23]. In short, the univariable association we observed most likely reflects a sicker patient phenotype rather than a direct pharmacological effect, and vasopressor requirement should be viewed as a marker of severity rather than a proximate cause of death. Nevertheless, given the observational design, residual confounding and indication bias cannot be ruled out.

Lactate is another biomarker that captures both tissue hypoperfusion and overall illness severity in SA-AKI. In a large cohort from Tang and colleagues, the lactate-to-albumin ratio was independently linked to higher 28-day mortality [24]. Wang and colleagues, in 717 SA-AKI cases (157 receiving continuous renal replacement therapy [CRRT]), found no association between CRRT use and 28-day mortality but did report an approximately 2.12-fold increase in mortality risk tied to a rising lactate trajectory over time [25]. Chen Lin and colleagues, in their retrospective analysis, reported that every 1 mmol/L rise in lactate carried roughly a 10.7% increase in 28-day mortality risk (HR: 1.107, *p* = 0.012) [26]. Our own univariable results echoed these patterns in both the overall cohort and the SA-AKI subset. On multivariable adjustment within the SA-AKI subgroup, each 1 mmol/L increment in lactate corresponded to about a 6% higher mortality risk, though the estimate fell just outside conventional statistical significance (HR = 1.06, 95% CI 1.00–1.13, *p* = 0.053). The size of this effect aligns well with the 10.7% figure reported by Chen Lin et al. [26], with a similar direction of association. However, since the confidence interval touches the null (1.00), we cannot claim statistical proof. We attribute this limitation to the sample size. The weaker but similarly directed trend in the whole cohort (HR = 1.05, 95% CI 0.99–1.11, *p* = 0.13) is consistent with this reading. Taken together, our findings support the concept of lactate as a biologically meaningful marker of both tissue perfusion and overall illness severity in SA-AKI, though larger, adequately powered prospective series are needed before confirming that lactate is independently associated with outcomes. In their large multicentre retrospective work, White and colleagues found the UO-only criterion to be the most common route to SA-AKI diagnosis, and this route came with milder AKI, better renal outcomes, and lower mortality [14]. Notably, they staged AKI at diagnosis, whereas we used the maximum stage attained within the first seven days after sepsis onset (as required by the SA-AKI definition). This methodological gap should be kept in mind: the higher proportion of stage 3 AKI among our SCr + UO cases probably reflects, in part, a more severe renal phenotype captured by the up-to-seven-day window. Consequently, direct comparisons of stage distribution with the diagnosis-time staging in White et al. would not be appropriate.

Several caveats apply to our study. First, it is a single-centre, retrospective analysis with a modest sample size, and generalisability is therefore constrained. Multivariable Cox modelling did retain SA-AKI as independently associated with 28-day mortality, but the retrospective design does not license causal claims. SA-AKI is therefore best regarded as a marker of a more severe septic phenotype featuring greater hemodynamic instability and organ dysfunction, rather than as a direct causal driver of death. Second, the modest sample restricts statistical power, particularly for mortality outcomes, and may have masked meaningful subgroup differences. Third, in the SA-AKI subgroup Cox model, the proportional hazards assumption was not fully met for CCI, SAPS II, or the SCr + UO pattern. Given these violations, the limited subgroup size, and the event-to-parameter ratio of 8.8, the adjusted subgroup estimates should be regarded as exploratory and hypothesis-generating. Fourth, our microbiological comparisons were exploratory and not corrected for multiple testing, and should be viewed cautiously and re-tested in larger prospective work. Finally, we captured only variables from the first 24 h after ICU admission; the impact of subsequent trajectory in clinical status, laboratory values, and management on the observed associations was not evaluated.

## 5. Conclusions

Roughly half of the septic ICU patients in this cohort developed SA-AKI. Its independent link with 28-day mortality survived adjustment for other clinical variables and was preserved across several sensitivity analyses. In contrast, once modelling was restricted to SA-AKI cases, no covariate reached significance in the multivariable step. Vasopressor use on day 1 was strongly associated with mortality in univariable analyses but weakened sharply after adjustment. Lactate fell just short of statistical significance, suggesting that it may still be a mortality-associated clinical marker in SA-AKI, although our sample may have been too small to demonstrate this association conclusively. Given the modest sample size and the methodological caveats of the subgroup analysis, our estimates must be read cautiously. On balance, the data indicate that SA-AKI is a clinically meaningful marker associated with higher short-term mortality in septic ICU patients. The observational design, however, requires that this link be seen as a strong association awaiting confirmation in prospective studies rather than as a causal relationship.

## Figures and Tables

**Table 1:** Baseline demographic, clinical, and laboratory characteristics according to SA-AKI development.

Characteristic	Total (*n* = 185)	SA-AKI Absent (*n* = 101)	SA-AKI Present (*n* = 84)	*p*-Value
Gender, *n* (%)				1.00
Female	79 (42.7)	43 (42.6)	36 (42.9)	
Male	106 (57.3)	58 (57.4)	48 (57.1)	
Age (years)	69.0 (58.0–77.0)	68.0 (58.0–78.0)	70.0 (60.8–77.0)	0.50
Septic shock, *n* (%)	74 (40.0)	23 (22.8)	51 (60.7)	<0.001
Source of ICU admission, *n* (%)				0.35
Emergency department	104 (56.2)	57 (56.4)	47 (56.0)	
Operating theatre	2 (1.1)	2 (2.0)	0 (0.0)	
Other hospital	5 (2.7)	4 (4.0)	1 (1.2)	
Ward	74 (40.0)	38 (37.6)	36 (42.9)	
Admission type, *n* (%)				0.73
Medical	172 (93.0)	95 (94.1)	77 (91.7)	
Surgical	13 (7.0)	6 (5.9)	7 (8.3)	
Nephrotoxic agent use, *n* (%)	26 (14.1)	1 (1.0)	25 (29.8)	<0.001
Comorbidities, *n* (%)				
Diabetes mellitus	56 (30.3)	29 (28.7)	27 (32.1)	0.73
Hypertension	76 (41.1)	35 (34.7)	41 (48.8)	0.072
Cardiovascular disease	35 (18.9)	13 (12.9)	22 (26.2)	0.034
Malignancy	64 (34.6)	25 (24.8)	39 (46.4)	0.003
COPD	37 (20.0)	24 (23.8)	13 (15.5)	0.22
Cerebrovascular disease	24 (13.0)	15 (14.9)	9 (10.7)	0.54
Chronic kidney disease	35 (18.9)	9 (8.9)	26 (31.0)	<0.001
Charlson Comorbidity Index	4.00 (3.00–6.00)	4.00 (2.00–5.00)	5.00 (3.00–9.25)	<0.001
Infection source, *n* (%)				
Lung	146 (78.9)	91 (90.1)	55 (65.5)	<0.001
Abdominal	18 (9.7)	4 (4.0)	14 (16.7)	0.005
Urinary tract	29 (15.7)	12 (11.9)	17 (20.2)	0.18
SSTI	9 (4.9)	2 (2.0)	7 (8.3)	0.081
Bacteraemia	45 (24.3)	15 (14.9)	30 (35.7)	0.002
CNS infection	1 (0.5)	0 (0.0)	1 (1.2)	0.45
Laboratory				
Platelets (10^3^/µL)	259 ± 152	285 ± 147	227 ± 153	0.010
pH	7.39 (7.27–7.45)	7.42 (7.35–7.48)	7.33 (7.20–7.43)	<0.001
Bicarbonate (mmol/L)	26.0 ± 8.3	29.1 ± 7.2	22.3 ± 8.0	<0.001
Lactate (mmol/L)	1.60 (1.10–2.40)	1.40 (1.00–1.80)	2.00 (1.30–4.78)	<0.001
Albumin (g/L)	27.2 ± 7.3	29.6 ± 7.6	24.3 ± 5.8	<0.001
Baseline creatinine (mg/dL)	0.76 (0.56–1.02)	0.67 (0.51–0.98)	0.80 (0.66–1.09)	0.007
MAP (mmHg)	83.3 (73.0–96.7)	89.0 (78.7–95.7)	78.2 (65.4–97.1)	0.006
Severity scores/early interventions				
SAPS II on ICU admission	44.9 ± 18.8	34.8 ± 13.4	57.0 ± 17.2	<0.001
First-day RRT	14 (7.6)	0 (0.0)	14 (16.7)	<0.001
First-day mechanical ventilation	87 (47.0)	40 (39.6)	47 (56.0)	0.038
First-day vasopressor	75 (40.5)	23 (22.8)	52 (61.9)	<0.001

Data are presented as *n* (%), mean ± standard deviation, or median (interquartile range). Continuous variables were compared with the Student t, Welch t, or Mann–Whitney U test as appropriate; categorical variables were compared with the χ^2^ or Fisher exact test. SA-AKI: sepsis-associated acute kidney injury, ICU: intensive care unit, COPD: chronic obstructive pulmonary disease, SSTI: skin and soft-tissue infection, CNS: central nervous system, MAP: mean arterial pressure, SAPS II: Simplified Acute Physiology Score II, RRT: renal replacement therapy.

**Table 2:** Clinical outcomes and microbiological findings according to SA-AKI development.

Variable	Total (*n* = 185)	SA-AKI Absent (*n* = 101)	SA-AKI Present (*n* = 84)	*p*-Value
AKI diagnostic pattern, *n* (%)				-
UO-only	16 (19.0)	NA	16 (19.0)	
SCr-only	23 (27.4)	NA	23 (27.4)	
SCr + UO	45 (53.6)	NA	45 (53.6)	
AKI stage, *n* (%)				-
Stage 1	20 (23.8)	NA	20 (23.8)	
Stage 2	16 (19.0)	NA	16 (19.0)	
Stage 3	48 (57.1)	NA	48 (57.1)	
ICU length of stay (days)	15.0 (6.0–32.2)	17.5 (8.0–36.2)	11.0 (5.0–22.2)	0.009
Hospital length of stay (days)	22.0 (12.0–41.5)	25.5 (14.8–43.8)	19.0 (10.0–33.2)	0.011
Vasopressor (any), *n* (%)	120 (64.9)	43 (42.6)	77 (91.7)	<0.001
RRT (any), *n* (%)	22 (11.9)	1 (1.0)	21 (25.0)	<0.001
Mechanical ventilation (any), *n* (%)	149 (80.5)	72 (71.3)	77 (91.7)	<0.001
Twenty-eight-day mortality, *n* (%)	71 (38.4)	18 (17.8)	53 (63.1)	<0.001
In-hospital mortality, *n* (%)	97 (52.4)	34 (33.7)	63 (75.0)	<0.001
Causative organism, *n* (%)				
Acinetobacter baumannii	15 (8.1)	9 (8.9)	6 (7.1)	0.87
Klebsiella pneumoniae	29 (15.7)	13 (12.9)	16 (19.0)	0.34
MSSA	12 (6.5)	6 (5.9)	6 (7.1)	0.98
MRSA	15 (8.1)	9 (8.9)	6 (7.1)	0.87
Enterococcus	5 (2.7)	2 (2.0)	3 (3.6)	0.66
Pseudomonas aeruginosa	19 (10.3)	13 (12.9)	6 (7.1)	0.30
Candida	3 (1.6)	2 (2.0)	1 (1.2)	1.00
Other Gram-negative	8 (4.3)	2 (2.0)	6 (7.1)	0.14
Escherichia coli	21 (11.4)	6 (5.9)	15 (17.9)	0.021

The AKI diagnostic pattern and AKI stage were assessed only in patients with SA-AKI. SCr: serum creatinine, UO: urine output, RRT: renal replacement therapy, MSSA/MRSA: methicillin-susceptible/resistant Staphylococcus aureus.

**Table 3:** Baseline demographic, clinical, and laboratory characteristics according to 28-day mortality.

Characteristic	Total (*n* = 185)	Alive (*n* = 114)	Dead (*n* = 71)	*p*-Value
Gender, *n* (%)				0.72
Female	79 (42.7)	47 (41.2)	32 (45.1)	
Male	106 (57.3)	67 (58.8)	39 (54.9)	
Age (years)	69.0 (58.0–77.0)	68.0 (57.2–77.0)	71.0 (61.0–78.0)	0.15
Septic shock, *n* (%)	74 (40.0)	35 (30.7)	39 (54.9)	0.002
Presence of SA-AKI, *n* (%)	84 (45.4)	31 (27.2)	53 (74.6)	<0.001
Source of ICU admission, *n* (%)				0.11
Emergency department	104 (56.2)	72 (63.2)	32 (45.1)	
Operating theatre	2 (1.1)	1 (0.9)	1 (1.4)	
Other hospital	5 (2.7)	2 (1.8)	3 (4.2)	
Ward	74 (40.0)	39 (34.2)	35 (49.3)	
Admission type, *n* (%)				1.00
Medical	172 (93.0)	106 (93.0)	66 (93.0)	
Surgical	13 (7.0)	8 (7.0)	5 (7.0)	
Nephrotoxic agent use, *n* (%)	26 (14.1)	7 (6.1)	19 (26.8)	<0.001
Comorbidities, *n* (%)				
Diabetes mellitus	56 (30.3)	37 (32.5)	19 (26.8)	0.51
Hypertension	76 (41.1)	42 (36.8)	34 (47.9)	0.18
Cardiovascular disease	35 (18.9)	19 (16.7)	16 (22.5)	0.42
Malignancy	64 (34.6)	28 (24.6)	36 (50.7)	<0.001
COPD	37 (20.0)	28 (24.6)	9 (12.7)	0.076
Cerebrovascular disease	24 (13.0)	17 (14.9)	7 (9.9)	0.44
Chronic kidney disease	35 (18.9)	12 (10.5)	23 (32.4)	<0.001
Charlson Comorbidity Index	4.00 (3.00–6.00)	4.00 (2.00–5.00)	5.00 (3.00–10.00)	<0.001
Infection source, *n* (%)				
Lung	146 (78.9)	94 (82.5)	52 (73.2)	0.19
Abdominal	18 (9.7)	8 (7.0)	10 (14.1)	0.19
Urinary tract	29 (15.7)	20 (17.5)	9 (12.7)	0.50
SSTI	9 (4.9)	4 (3.5)	5 (7.0)	0.31
Bacteraemia	45 (24.3)	24 (21.1)	21 (29.6)	0.26
CNS infection	1 (0.5)	0 (0.0)	1 (1.4)	0.38
Laboratory				
Platelets (10^3^/µL)	259 ± 152	283 ± 155	219 ± 140	0.005
pH	7.39 (7.27–7.45)	7.41 (7.27–7.46)	7.36 (7.25–7.44)	0.099
Bicarbonate (mmol/L)	26.0 ± 8.3	26.5 ± 8.1	25.2 ± 8.6	0.31
Lactate (mmol/L)	1.60 (1.10–2.40)	1.40 (1.00–1.90)	2.10 (1.30–4.45)	<0.001
Albumin (g/L)	27.2 ± 7.3	28.8 ± 7.7	24.5 ± 5.7	<0.001
Baseline creatinine (mg/dL)	0.76 (0.56–1.02)	0.73 (0.55–1.02)	0.77 (0.58–0.99)	0.68
Serum creatinine at ICU admission (mg/dL)	1.06 (0.66–2.06)	0.93 (0.61–1.83)	1.48 (0.88–2.42)	0.002
Daily urine output (mL)	1561 ± 912	1826 ± 805	1145 ± 919	<0.001
Mean hourly urine output (mL/kg/h)	0.87 ± 0.51	1.00 ± 0.45	0.66 ± 0.54	<0.001
MAP (mmHg)	83.3 (73.0–96.7)	88.3 (75.4–95.7)	78.7 (70.7–98.2)	0.059
Severity scores/early interventions				
SAPS II on ICU admission	44.9 ± 18.8	38.4 ± 16.6	55.4 ± 17.5	<0.001
First-day RRT	14 (7.6)	3 (2.6)	11 (15.5)	0.003
First-day mechanical ventilation	87 (47.0)	49 (43.0)	38 (53.5)	0.21
First-day vasopressor	75 (40.5)	35 (30.7)	40 (56.3)	<0.001

Data are presented as *n* (%), mean ± standard deviation, or median (interquartile range). SA-AKI: sepsis-associated acute kidney injury, CCI: Charlson Comorbidity Index, MAP: mean arterial pressure.

**Table 4:** Univariable and multivariable Cox proportional hazards analyses of factors associated with 28-day mortality in the overall cohort.

Variable	Univariable HR (95% CI)	*p*-Value	Multivariable HR (95% CI)	*p*-Value
Age (per 10 years)	1.11 (0.95–1.30)	0.18	0.99 (0.83–1.18)	0.90
Male sex	0.92 (0.58–1.47)	0.73		
Presence of SA-AKI	5.45 (3.18–9.33)	<0.001	2.87 (1.51–5.45)	0.001
Septic shock	2.28 (1.43–3.65)	<0.001		
Nephrotoxic agent use	3.62 (2.13–6.16)	<0.001		
Diabetes mellitus	0.78 (0.46–1.32)	0.35		
Hypertension	1.41 (0.89–2.25)	0.15		
Cardiovascular disease	1.36 (0.78–2.37)	0.28		
Malignancy	2.44 (1.53–3.89)	<0.001		
COPD	0.52 (0.26–1.04)	0.066		
CKD	2.76 (1.67–4.55)	<0.001		
Charlson Comorbidity Index (per 1 point)	1.15 (1.08–1.22)	<0.001	1.06 (0.98–1.13)	0.14
Lactate (per 1 mmol/L)	1.14 (1.08–1.20)	<0.001	1.05 (0.99–1.11)	0.12
Albumin (per 1 g/L)	0.95 (0.92–0.97)	<0.001	0.97 (0.94–1.01)	0.14
MAP (per 10 mmHg)	1.01 (0.93–1.09)	0.90		
SAPS II (per 10 points)	1.50 (1.33–1.69)	<0.001	1.18 (0.98–1.40)	0.074
First-day RRT	2.91 (1.53–5.54)	0.001		
First-day mechanical ventilation	1.44 (0.90–2.30)	0.12		
First-day vasopressor	2.41 (1.51–3.86)	<0.001	1.09 (0.63–1.89)	0.75

The multivariable model was pre-specified based on clinical rationale: presence of SA-AKI (exposure), age, Charlson Comorbidity Index, lactate, SAPS II, albumin, and first-day vasopressor. CKD, malignancy, and diabetes are represented within the CCI (to avoid collinearity). Septic shock is represented via SAPS II and vasopressor. Nephrotoxic exposure and first-day RRT were not included in the model as potential mediators on the SA-AKI → mortality pathway. *n* = 185, events = 71, event-to-variable ratio = 10.1. Multicollinearity: all VIFs ranged from 1.20 to 2.53 (threshold < 5). Harrell C-index = 0.773. The proportional hazards assumption was met for all covariates except albumin (*p* = 0.040, borderline). HR: hazard ratio, CI: confidence interval.

**Table 5:** Univariable and multivariable Cox proportional hazards analyses of factors associated with 28-day mortality—SA-AKI subgroup (*n* = 84).

Variable	Univariable HR (95% CI)	*p*-Value	Multivariable HR (95% CI)	*p*-Value
Age (per 10 years)	1.09 (0.89–1.33)	0.42		
Male sex	1.08 (0.63–1.86)	0.78		
Septic shock	1.81 (1.01–3.22)	0.045		
Nephrotoxic agent use	1.75 (1.00–3.08)	0.051		
Malignancy	1.98 (1.14–3.42)	0.015		
CKD	1.87 (1.08–3.26)	0.027		
Charlson Comorbidity Index (per 1 point)	1.09 (1.01–1.16)	0.018	1.05 (0.98–1.13)	0.16
Lactate (per 1 mmol/L)	1.08 (1.02–1.14)	0.010	1.06 (1.00–1.13)	0.053
Albumin (per 1 g/L)	0.97 (0.92–1.01)	0.18		
SAPS II (per 10 points)	1.27 (1.09–1.49)	0.003	1.13 (0.92–1.37)	0.25
AKI diagnostic pattern				
UO-only (reference)	Reference	-	Reference	-
SCr-only vs. UO-only	0.72 (0.38–1.34)	0.29	0.80 (0.35–1.84)	0.60
SCr + UO vs. UO-only	1.16 (0.68–2.00)	0.59	0.63 (0.30–1.31)	0.21
AKI stage				
Stage 1 (reference)	Reference	-		
Stage 2 vs. Stage 1	0.55 (0.25–1.22)	0.14		
Stage 3 vs. Stage 1	1.05 (0.61–1.80)	0.87		
First-day RRT	1.29 (0.66–2.51)	0.45		
First-day mechanical ventilation	1.02 (0.59–1.76)	0.94		
First-day vasopressor	1.96 (1.09–3.53)	0.025	1.61 (0.82–3.14)	0.16

Multivariable model in SA-AKI patients: CCI, lactate, SAPS II, first-day vasopressor, and AKI diagnostic pattern (UO-only reference). *n* = 84, events = 53, event-to-variable ratio = 8.8. Harrell C-index = 0.664. SCr: serum creatinine, UO: urine output.

## Data Availability

The data that support the findings of this study are available on request from the corresponding author, İ.K. The data are not publicly available due to ethical and institutional restrictions related to patient confidentiality.

## Supplementary Materials

The following supporting information can be downloaded at: https://www.mdpi.com/article/10.3390/jcm15166171/s1, Supplementary Table S1: Sensitivity multivariable Cox model with MDRD-imputed baseline creatinine as a covariate, Supplementary Table S2: Clinical outcomes and microbiological findings according to 28-day mortality, Supplementary Table S3: Proportional-hazards assumption testing (scaled Schoenfeld residuals) for the primary and SA-AKI subgroup Cox models, Supplementary Table S4: Sensitivity analyses of the SA-AKI–mortality association across model specifications, Supplementary Table S5: Baseline characteristics, interventions and outcomes according to AKI diagnostic pattern in patients with SA-AKI, Supplementary Table S6: Twenty-eight-day mortality by AKI diagnostic pattern with 95% confidence intervals (exploratory), Supplementary Figure S1: Forest plot of SA-AKI hazard ratios for 28-day mortality across five model specifications.

## References

[1] Hoste E.A.J., Bagshaw S.M., Bellomo R., Cely C.M., Colman R., Cruz D.N., Edipidis K., Forni L.G., Gomersall C.D., Govil D. (2015). Epidemiology of acute kidney injury in critically ill patients: The multinational AKI-EPI study. Intensive Care Med..

[2] Uchino S., Kellum J.A., Bellomo R., Doig G.S., Morimatsu H., Morgera S., Schetz M., Tan I., Bouman C., Macedo E. (2005). Acute renal failure in critically ill patients: A multinational, multicenter study. JAMA.

[3] Peerapornratana S., Manrique-Caballero C.L., Gómez H., Kellum J.A. (2019). Acute kidney injury from sepsis: Current concepts, epidemiology, pathophysiology, prevention and treatment. Kidney Int..

[4] Rudd K.E., Johnson S.C., Agesa K.M., Shackelford K.A., Tsoi D., Kievlan D.R., Colombara D.V., Ikuta K.S., Kissoon N., Finfer S. (2020). Global, regional, and national sepsis incidence and mortality, 1990–2017: Analysis for the Global Burden of Disease Study. Lancet.

[5] Song M.J., Jang Y., Legrand M., Park S., Ko R.E., Suh G.Y., Oh D.K., Lee S.Y., Park M.H., Lim C.M. (2024). Epidemiology of sepsis-associated acute kidney injury in critically ill patients: A multicenter, prospective, observational cohort study in South Korea. Crit. Care.

[6] Takeuchi T., Flannery A.H., Liu L.J., Ghazi L., Cama-Olivares A., Fushimi K., Chen J., Huen S.C., Tolwani A.J., Neyra J.A. (2025). Epidemiology of sepsis-associated acute kidney injury in the ICU with contemporary consensus definitions. Crit. Care.

[7] Zarbock A., Nadim M.K., Pickkers P., Gomez H., Bell S., Joannidis M., Kashani K., Koyner J.L., Pannu N., Meersch M. (2023). Sepsis-associated acute kidney injury: Consensus report of the 28th Acute Disease Quality Initiative workgroup. Nat. Rev. Nephrol..

[8] Singer M., Deutschman C.S., Seymour C.W., Shankar-Hari M., Annane D., Bauer M., Bellomo R., Bernard G.R., Chiche J.D., Coopersmith C.M. (2016). The Third International Consensus Definitions for Sepsis and Septic Shock (Sepsis-3). JAMA.

[9] Seymour C.W., Liu V.X., Iwashyna T.J., Brunkhorst F.M., Rea T.D., Scherag A., Rubenfeld G., Kahn J.M., Shankar-Hari M., Singer M. (2016). Assessment of Clinical Criteria for Sepsis: For the Third International Consensus Definitions for Sepsis and Septic Shock (Sepsis-3). JAMA.

[10] Raith E.P., Udy A.A., Bailey M., McGloughlin S., MacIsaac C., Bellomo R., Pilcher D.V. (2017). Prognostic Accuracy of the SOFA Score, SIRS Criteria, and qSOFA Score for In-Hospital Mortality Among Adults With Suspected Infection Admitted to the Intensive Care Unit. JAMA.

[11] Lambden S., Laterre P.F., Levy M.M., François B. (2019). The SOFA score—Development, utility and challenges of accurate assessment in clinical trials. Crit. Care.

[12] Shankar-Hari M., Phillips G.S., Levy M.L., Seymour C.W., Liu V.X., Deutschman C.S., Angus D.C., Rubenfeld G.D., Singer M. (2016). Developing a New Definition and Assessing New Clinical Criteria for Septic Shock: For the Third International Consensus Definitions for Sepsis and Septic Shock (Sepsis-3). JAMA.

[13] Khwaja A. (2012). KDIGO clinical practice guidelines for acute kidney injury. Nephron Clin. Pract..

[14] White K.C., Serpa-Neto A., Hurford R., Clement P., Laupland K.B., See E., McCullough J., White H., Shekar K., Tabah A. (2023). Sepsis-associated acute kidney injury in the intensive care unit: Incidence, patient characteristics, timing, trajectory, treatment, and associated outcomes. Intensive Care Med..

[15] (2024). Kidney Disease: Improving Global Outcomes (KDIGO) CKD Work Group. KDIGO 2024 Clinical Practice Guideline for the Evaluation and Management of Chronic Kidney Disease. Kidney Int..

[16] Donaldson L.H., Vlok R., Sakurai K., Burrows M., McDonald G., Venkatesh K., Bagshaw S.M., Bellomo R., Delaney A., Myburgh J. (2024). Quantifying the Impact of Alternative Definitions of Sepsis-Associated Acute Kidney Injury on Its Incidence and Outcomes: A Systematic Review and Meta-Analysis. Crit. Care Med..

[17] Liu J., Xie H., Ye Z., Li F., Wang L. (2020). Rates, predictors, and mortality of sepsis-associated acute kidney injury: A systematic review and meta-analysis. BMC Nephrol..

[18] Rusu M., Ichim C., Anderco P., Pălăștea A., Boicean A. (2026). Gut-Kidney Axis: Unraveling the Role of the Microbiome in Chronic Kidney Disease. Biomedicines.

[19] Bai X., Yu W., Ji W., Lin Z., Tan S., Duan K., Dong Y., Xu L., Li N. (2014). Early versus delayed administration of norepinephrine in patients with septic shock. Crit. Care.

[20] Asfar P., Meziani F., Hamel J.F., Grelon F., Megarbane B., Anguel N., Mira J.P., Dequin P.F., Gergaud S., Weiss N. (2014). High versus low blood-pressure target in patients with septic shock. N. Engl. J. Med..

[21] Zeng J., Zhang Y., Zhang M., Feng K., Guo W., Ge Y., Chen W., Li J., Cheng A., Bai J. (2025). Analysis of prognostic risk factors in critically ill elderly patients with sepsis-associated acute kidney injury. BMC Nephrol..

[22] Calzavacca P., Evans R.G., Bailey M., Bellomo R., May C.N. (2015). Variable responses of regional renal oxygenation and perfusion to vasoactive agents in awake sheep. Am. J. Physiol. Regul. Integr. Comp. Physiol..

[23] Lankadeva Y.R., Kosaka J., Evans R.G., Bailey S.R., Bellomo R., May C.N. (2016). Intrarenal and urinary oxygenation during norepinephrine resuscitation in ovine septic acute kidney injury. Kidney Int..

[24] Tang H., Qu M., Xin M., He T. (2025). Association between lactate-albumin ratio and 28-day mortality in patients with sepsis-associated acute kidney injury: A retrospective cohort study. Sci. Rep..

[25] Wang Z., Zhang L., Xu F., Han D., Lyu J. (2022). The association between continuous renal replacement therapy as treatment for sepsis-associated acute kidney injury and trend of lactate trajectory as risk factor of 28-day mortality in intensive care units. BMC Emerg. Med..

[26] Lin C., Wang J., Cai K., Luo Y., Wu W., Lin S., Lin Z., Feng S. (2024). Elevated Activated Partial Thromboplastin Time as a Predictor of 28-Day Mortality in Sepsis-Associated Acute Kidney Injury: A Retrospective Cohort Analysis. Int. J. Gen. Med..

