# Supplementary material for: Sepsis-Associated Acute Kidney Injury and 28-Day Mortality in Critically Ill Patients with Sepsis: A Retrospective Cohort Study
Source: J Clin Med. 2026 Aug 9;15(16):6171. doi: 10.3390/jcm15166171 (PMC13513228; doi:10.3390/jcm15166171)
Supplement: Supplementary file 1 [file jcm-15-06171-s001.zip › jcm-4448371-supplementary.pdf]

SUPPLEMENTARY MATERIALS

Supplementary Table S1. Sensitivity multivariable Cox model with MDRD-imputed baseline creatinine as a covariate.

Covariate	HR (95% CI)	p-value
Presence of SA-AKI	2.83 (1.49-5.41)	0.002
Age (per 10 years)	1.00 (0.83-1.19)	0.98
Charlson Comorbidity Index (per 1 point)	1.05 (0.98-1.13)	0.16
Lactate (per 1 mmol/L)	1.05 (0.99-1.11)	0.11
SAPS II (per 10 points)	1.18 (0.99-1.41)	0.065
Albumin (per 1 g/L)	0.97 (0.94-1.01)	0.17
First-day vasopressor	1.07 (0.62-1.86)	0.81
Baseline creatinine, MDRD-imputed (per 1 mg/dL)	0.89 (0.56-1.40)	0.61

N=185, events=71. Harrell's C-index=0.776. Baseline creatinine was documented for 178/185 patients (96.2%); MDRD back-estimation (assuming eGFR = 75 mL/min/1.73 m²) was applied for the remaining 7 patients (3.8%). The primary SA-AKI hazard ratio is essentially unchanged (2.83 vs. 2.87 in Table 4).

Supplementary Table S2. Clinical outcomes and microbiological findings according to 28-day mortality.

Variable	Total (N=185)	Alive (n=114)	Dead (n=71)	p-value
ICU length of stay (days)	15.0 (6.0-32.2)	23.0 (9.2-49.5)	10.0 (4.0-15.0)	<0.001
Hospital length of stay (days)	22.0 (12.0-41.5)	32.5 (16.0-54.8)	14.5 (9.0-23.0)	<0.001
Vasopressor (any), n (%)	120 (64.9)	52 (45.6)	68 (95.8)	<0.001
RRT (any), n (%)	22 (11.9)	5 (4.4)	17 (23.9)	<0.001
Mechanical ventilation (any), n (%)	149 (80.5)	79 (69.3)	70 (98.6)	<0.001
In-hospital mortality, n (%)	97 (52.4)	26 (22.8)	71 (100.0)	<0.001
**Causative organism, n (%)**				
Acinetobacter baumannii	15 (8.1)	10 (8.8)	5 (7.0)	0.89
Klebsiella pneumoniae	29 (15.7)	13 (11.4)	16 (22.5)	0.069
MSSA	12 (6.5)	10 (8.8)	2 (2.8)	0.13
MRSA	15 (8.1)	7 (6.1)	8 (11.3)	0.33
Enterococcus	5 (2.7)	2 (1.8)	3 (4.2)	0.37
Pseudomonas aeruginosa	19 (10.3)	11 (9.6)	8 (11.3)	0.92
Candida	3 (1.6)	2 (1.8)	1 (1.4)	1.00
Other gram-negative	8 (4.3)	5 (4.4)	3 (4.2)	1.00
Escherichia coli	21 (11.4)	14 (12.3)	7 (9.9)	0.79

RRT: *renal replacement therapy.*

Supplementary Table S3. Proportional-hazards assumption testing (scaled Schoenfeld residuals) for the primary and SA-AKI subgroup Cox models.

Model	Covariate	χ^2 statistic	p-value	Interpretation
**Primary Cox (overall cohort)**				
	Presence of SA-AKI	1.79	0.181	PH satisfied
	Age (per 10 years)	0.29	0.591	PH satisfied
	Charlson Comorbidity Index	2.38	0.123	PH satisfied
	Lactate	2.46	0.117	PH satisfied
	SAPS II (per 10 points)	2.05	0.152	PH satisfied
	Albumin	4.21	0.040	Borderline
	First-day vasopressor	0.01	0.919	PH satisfied
**SA-AKI subgroup Cox**				
	Charlson Comorbidity Index	4.42	0.035	PH violated
	Lactate	0.00	0.975	PH satisfied
	SAPS II (per 10 points)	4.25	0.039	PH violated
	First-day vasopressor	0.02	0.881	PH satisfied
	AKI pattern: SCr-only vs UO-only	2.34	0.126	PH satisfied
	AKI pattern: SCr + UO vs UO-only	10.19	0.001	PH violated

The proportional-hazards assumption was tested using scaled Schoenfeld residuals with time transformed by rank. p-values > 0.05 support the assumption. In the SA-AKI subgroup model, three covariates (CCI, SAPS II, SCr+UO pattern) showed evidence of non-proportional hazards; results in Table 5 should be interpreted accordingly.

Supplementary Table S4. Sensitivity analyses of the SA-AKI–mortality association across model specifications.

Analysis	N	Events	SA-AKI HR (95% CI)	p-value	C-index
Primary multivariable Cox (full cohort)	185	71	2.87 (1.51-5.45)	0.001	0.773
Baseline-restricted (SA-AKI at day 0 only)	163	57	2.86 (1.42-5.74)	0.003	0.779
Landmark analysis at day 7	159	45	2.82 (1.33-6.00)	0.007	0.752
Time-dependent Cox (SA-AKI as time-varying)	185	71	2.71 (1.44-5.10)	0.002	—
MDRD-imputed baseline creatinine	185	71	2.83 (1.49-5.41)	0.002	0.776

All models adjusted for age (per 10 years), Charlson Comorbidity Index, lactate, SAPS II (per 10 points), albumin, and first-day vasopressor. The baseline-restricted analysis excluded patients whose SA-AKI onset was after ICU day 0. The landmark analysis included only patients alive at day 7. The time-dependent model treated SA-AKI as a time-varying binary exposure using a long-format survival dataset. The MDRD sensitivity analysis imputed

baseline creatinine using the MDRD equation (eGFR = 75 mL/min/1.73 m²) for the 7 patients without a documented pre-hospital baseline.

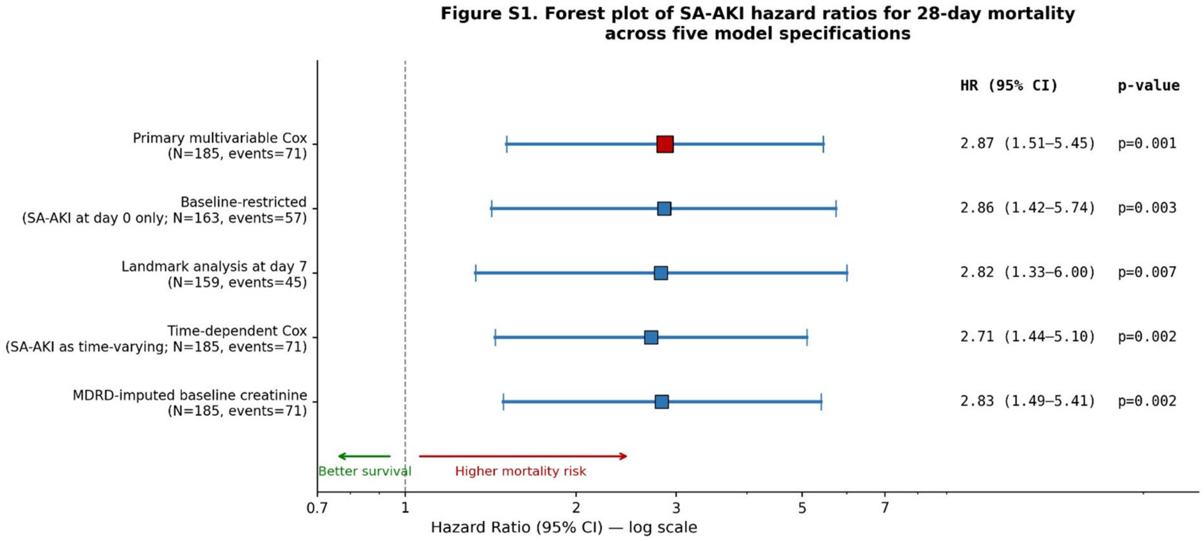

Figure S1. Forest plot of SA-AKI hazard ratios for 28-day mortality across five model specifications

Supplementary Table S5. Baseline characteristics, interventions and outcomes according to AKI diagnostic pattern in patients with SA-AKI.

Variable	UO-only (n=16)	SCr-only (n=23)	SCr + UO (n=45)	p-value
Age (years)	69.8 ± 16.1	66.8 ± 12.6	67.3 ± 14.9	0.80
Gender, n (%)				0.061
Female	3 (18.8)	13 (56.5)	20 (44.4)	
Male	13 (81.2)	10 (43.5)	25 (55.6)	
Septic shock, n (%)	6 (37.5)	11 (47.8)	34 (75.6)	0.009
Nephrotoxic agent use, n (%)	2 (12.5)	7 (30.4)	16 (35.6)	0.22
CKD, n (%)	4 (25.0)	5 (21.7)	17 (37.8)	0.34
Charlson Comorbidity Index	5.75 ± 3.55	5.26 ± 3.63	6.20 ± 3.95	0.62
Lactate (mmol/L)	1.80 (1.20-2.80)	1.80 (1.00-2.10)	2.40 (1.60-7.00)	0.010
Albumin (g/L)	23.3 ± 5.4	24.6 ± 4.5	24.5 ± 6.5	0.74
Baseline creatinine (mg/dL)	0.60 (0.51-0.76)	0.98 (0.78-1.10)	0.82 (0.68-1.12)	0.003
Serum creatinine at ICU admission (mg/dL)	1.02 (0.75-1.15)	2.04 (1.61-2.67)	2.29 (1.71-3.06)	<0.001
MAP (mmHg)	83.8 (63.9-99.6)	77.7 (66.3-97.3)	77.7 (65.7-94.7)	0.94
SAPS II	56.6 ± 16.8	44.9 ± 17.1	63.4 ± 14.0	<0.001
AKI stage, n (%)				0.001
Stage 1	4 (25.0)	12 (52.2)	4 (8.9)	
Stage 2	5 (31.2)	3 (13.0)	8 (17.8)	
Stage 3	7 (43.8)	8 (34.8)	33 (73.3)	
First-day mechanical ventilation, n (%)	7 (43.8)	10 (43.5)	30 (66.7)	0.10
First-day vasopressor, n (%)	6 (37.5)	11 (47.8)	35 (77.8)	0.005

First-day RRT, n (%)	2 (12.5)	1 (4.3)	11 (24.4)	0.097
ICU length of stay (days)	13.5 (8.8-18.2)	10.0 (6.5-17.0)	9.00 (3.00-32.00)	0.54
Hospital length of stay (days)	21.5 (12.2-30.2)	15.0 (10.5-20.5)	20.0 (9.0-37.0)	0.62
28-day mortality, n (%)	12 (75.0)	13 (56.5)	28 (62.2)	0.49
In-hospital mortality, n (%)	14 (87.5)	14 (60.9)	35 (77.8)	0.14

These subgroup comparisons are exploratory. Sample sizes are small; findings should be interpreted as hypothesis-generating. Continuous variables: one-way ANOVA or Kruskal–Wallis as appropriate. Categorical: χ^2 or Fisher's exact. **UO:** urine output; **SCr:** serum creatinine; **MAP:** mean arterial pressure; **SAPS II:** Simplified Acute Physiology Score II; **RRT:** renal replacement therapy.

Supplementary Table S6. Twenty-eight-day mortality by AKI diagnostic pattern with 95% confidence intervals (exploratory).

Pattern	n	Deaths, n (%)	95% CI for mortality	Unadjusted HR (95% CI)	p-value
UO-only	16	12 (75.0%)	50.5–89.8%	Reference	-
SCr-only	23	13 (56.5%)	36.8–74.4%	0.68 (0.31-1.50)	0.34
SCr + UO	45	28 (62.2%)	47.6–74.9%	0.93 (0.47-1.85)	0.84

Wilson 95% confidence intervals for mortality proportions. Cox regression compared each pattern with UO-only as the reference. Subgroup sizes are small (16–45 per group); confidence intervals overlap. These analyses are exploratory and hypothesis-generating.
